# Rural development and shifts in household dietary practices from 1999 to 2010 in the Tapajós River region, Brazilian Amazon: empirical evidence from dietary surveys

**DOI:** 10.1186/s12992-020-00564-5

**Published:** 2020-04-22

**Authors:** Jordan Sky Oestreicher, Deusilene Pereira do Amaral, Carlos José Sousa Passos, Myriam Fillion, Donna Mergler, Robert Davidson, Marc Lucotte, Christina A. Romaña, Frédéric Mertens

**Affiliations:** 1 Centro de Desenvolvimento Sustentável, Universidade de Brasília, Campus Universitário Darcy Ribeiro, Gleba A, Asa Norte, Brasília, DF 70910-900 Brazil; 2 Université TÉLUQ, Montréal, Canada; 3 Université du Québec à Montréal, Montréal, Canada; 4 Biodôme de Montréal, Montréal, Canada; 5 Université Paris Descartes, Paris, France

**Keywords:** Traditional diet, Amazon, Nutrition transition, Community, Rural development, Alimentação tradicional, Amazônia, transição alimentar, comunidade, desenvolvimento rural

## Abstract

**Background:**

Research on changing dietary practices is rare in lower and middle income countries, and understanding the impact of global economic processes on population health and nutrition is important, especially of rural communities. We analyzed the diet of 22 families in Brasília Legal, a riverside community in the Tapajós River region of the Brazilian Amazon, using nonparametric tests to compare dietary surveys taken in 1999 and 2010.

**Results:**

Data from the two surveys show that food obtained through commercial supply chains became more frequent in household diets, corresponding to significant increases in daily consumption of food items rich in energy, protein, and sugar. At the same time, there was a decline in traditional Amazonian food intake.

**Conclusions:**

Comparing these results with household socio-economic characteristics and drawing on open-ended interviews, we consider the multiple influences that economic development processes may have had on local diets. The introduction of new income sources and employment opportunities, infrastructural and transportation expansion, as well as environmental change appear to have influenced the observed dietary shifts. Such shifts are likely to have important implications for the nutritional status of communities in the Amazon, highlighting concerning trade-offs between current development trajectories and human health. Public policies and health education programs must urgently consider the interactions between sustainable development priorities in order to address emerging health risks in this rapidly changing region.

## Portuguese abstract

**Antecedentes:**

Pesquisas sobre mudanças nas práticas alimentares são raras nos países de renda média e baixa, mas são necessárias no intuito de compreender o impacto dos processos econômicos globais na saúde e nutrição das populações, especialmente em comunidades rurais. Utilizando-se inquéritos alimentares, analisou-se o consumo alimentar de 22 famílias em Brasília Legal, uma comunidade ribeirinha da região do rio Tapajós na Amazônia brasileira, usando-se testes não paramétricos para comparar dados de inquéritos alimentares realizados em 1999 e 2010.

**Resultados:**

Os dados dos dois inquéritos mostram que os alimentos obtidos por meio da cadeia comercial de suprimentos se tornaram mais frequentes nos perfis de consumo doméstico, levando a aumentos significativos no consumo diário de alimentos ricos em energia, proteína e açúcar. Ao mesmo tempo, houve um declínio na ingestão de alimentos tradicionais da Amazônia.

**Conclusões:**

Comparando-se esses resultados com as características socioeconômicas das famílias e baseando-se em entrevistas abertas, consideram-se as múltiplas influências que os processos de desenvolvimento econômico podem ter tido nos perfis de consumo alimentar local. A introdução de novas fontes de renda e oportunidades de emprego, a expansão de infraestruturas e do transporte, bem como as mudanças ambientais parecem ter influenciado as mudanças alimentares observadas. É provável que essas mudanças tenham implicações importantes para o estado nutricional das comunidades na Amazônia, destacando-se possíveis impactos preocupantes das atuais trajetórias de desenvolvimento sobre a saúde humana. As políticas públicas e os programas de educação em saúde devem considerar urgentemente as interações entre as diversas prioridades de desenvolvimento sustentável, a fim de enfrentar os riscos emergentes à saúde nessa região em rápida transformação.

## Introduction

Human history has been marked by food and nutritional changes, influenced by a range of environmental, economic, geographic and social factors. Over the last three centuries, dietary habits and nutritional status changed rapidly in North America and Europe, notably after World War II [1–3]. More recently, globalization processes that affect the availability, type, cost, and desirability of foods have triggered transitions throughout the developing world [4, 5]. According to the Food and Agriculture Organization (FAO), nutrition transitions are characterized by both quantitative and qualitative shifts in food consumption behavior and patterns [6]. These include increased intake of saturated fats (mainly from animal sources) and reduced intake of complex carbohydrates and fibers, as well as adverse changes in food structure leading to the consumption of foods with higher energy density and higher levels of added fats and sugars [7]. Such consumption patterns emerge with changes in household income, food prices, cultural traditions, and individual beliefs and preferences [6].

In Latin America, traditional diets based on foods that are rich in fibers, complex carbohydrates, trace elements, and phytochemical compounds have been replaced by diets that are characterized by refined sugars, animal products, and highly processed foods [8, 9]. Linked to globalization and the expansion of the neoliberal market economy, this transition has been so rapid in many countries that both excessive protein intake and malnutrition coexist. Paradoxically, the prevalence of obesity increases, while people still have insufficient food resources associated with undernutrition – a phenomenon known as “the double burden of malnutrition” [8, 10].

In Brazil, the seminal book *Geografia da Fome* (Geography of Hunger) was the first to consolidate and systematize national information on food and nutrition [11]. The ground-breaking work painted a broad portrait of food and nutrition disparities across the country by grouping Brazil into three geographic regions: endemic hunger in the Amazon and northeastern forested region, epidemic hunger in the semi-arid northeastern region, and malnutrition/hidden hunger in central and southern Brazil. In the first edition of the book, published in 1946, the available anthropometric, clinical and biochemical indicators were neither sufficiently consistent nor standardized to assess nutritional status at the epidemiological level [12]. It wasn’t until 1975 that Brazil’s different geographic regions were considered in national nutritional surveys [12].

In the Brazilian Amazon, dietary practices are strongly influenced by the regional biogeography, indigenous traditions, and the presence of European colonists and African descendants [13]. Combined with popular religious beliefs and food taboos, these factors have led to a distinct regional diet and a diversity of local eating habits. In riverside communities of mixed ancestry, dietary practices are shaped by seasonal climate and market variations, social representation and class, as well as individual and cultural preferences [14]. Diversified local economies based on fishing, hunting, slash-and-burn agriculture, and the extraction and sale of forest products have been practiced for centuries in these communities and form the basis of their daily diets [15]. Some recent studies have found evidence of dietary changes in the rural Brazilian Amazon that are indicative of a nutrition transition (c.f [5, 9, 16–18].). While they employ a variety of innovative methods, a comparison of dietary practices over time in the same sample population has, to our knowledge, not yet been undertaken.

In Brasília Legal, a rural riverside community in the lower Tapajós River region in western Pará state, a dietary survey was conducted in 1999 as part of a larger interdisciplinary research project [19]. The daily food intake of 26 families was evaluated using a dietary survey containing a list of key foods [20]. The study demonstrated that households had access to a diversity of foods, suggesting that families were probably meeting the minimum nutritional requirements for maintaining good health. Since then, political agendas to modernize the national agri-food industry and global demand for commodities such as soy and beef have had a stronger-than-ever effect on local land and food systems, with contentious deforestation and agribusiness expansion continuing to affect the viability of traditional systems based on fishing, hunting, and gathering [21, 22]. Changes in community demographics and economic relationships have been triggered by urbanization, changing access to external markets, and the introduction of social programs subsidizing household incomes [21, 22]. These localized impacts of economic development, linked to globalization and neoliberal policies, thus represent an important potential influence on nutritional status of populations [2, 4, 6].

In this paper, we conducted a follow-up dietary survey with the same families evaluated by Passos et al. [20]. Our objective was to compare dietary data collected in 1999 with new data, collected in 2010, and to examine corresponding changes in living standards and household socio-economic characteristics. Such methodologies are appropriate for evaluating diet-health relationships in places where globalized economic processes are rapidly changing local ways of life [18, 23]. Comparisons of dietary habits over time is rare in lower and middle income countries and is especially uncommon in rural areas due to challenges of working in remote regions. Longitudinal population health studies are also time consuming and expensive in these settings, yet they are necessary to identify food-related health risks that may be emerging from rapid economic transitions.

## Methods

### Study area

**Fig. 1:**
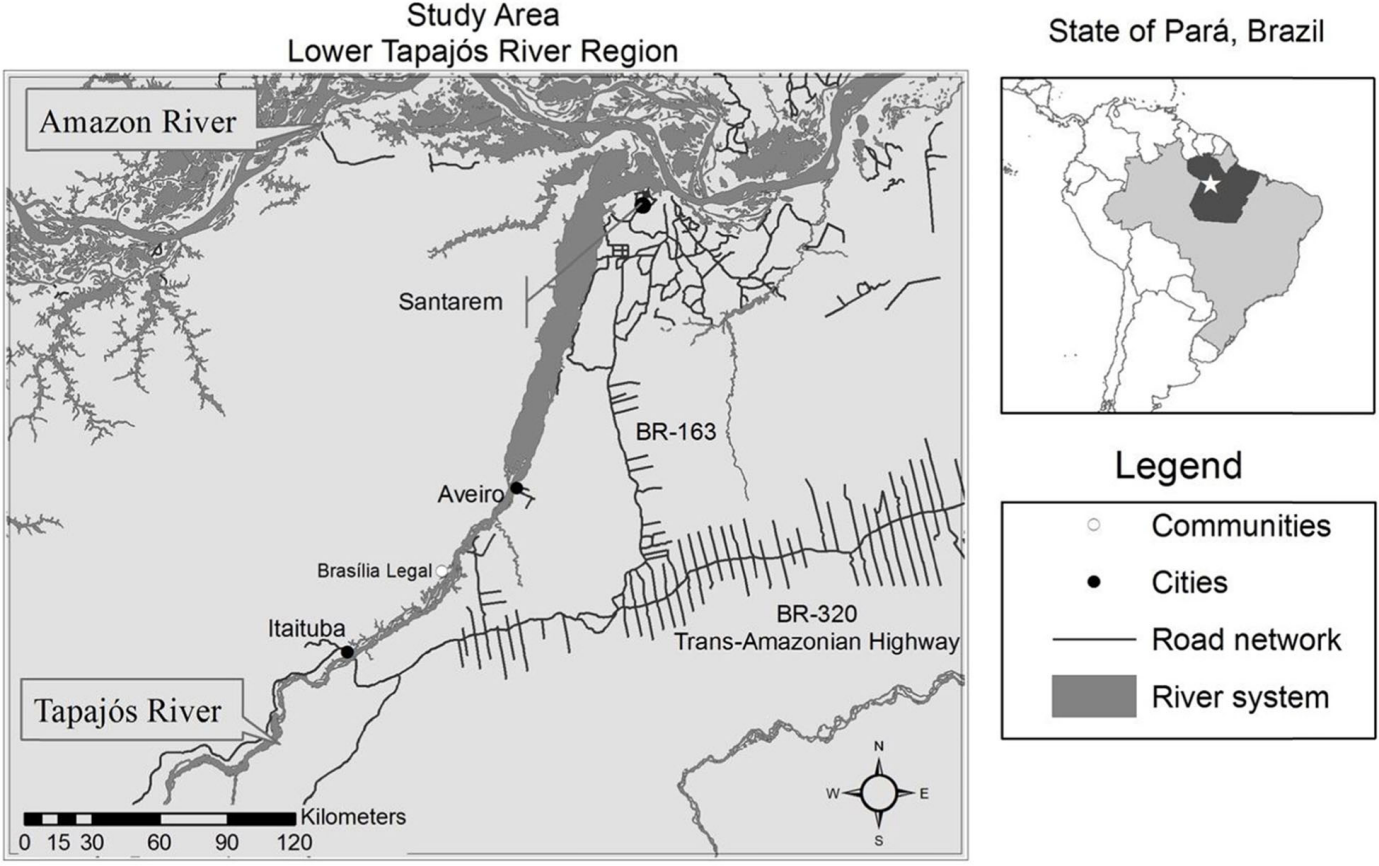
Tapajos River region and location of the community Brasília Legal

This study was conducted in the community of Brasília Legal, located in the municipality of Aveiro (Fig. 1). The village was established in 1836 as a center of resistance and trading post during the *Cabanagem* social movement [24]. Over the next 150 years, the population dynamics and economic relationships in the region were influenced by the rubber boom (1890 – 1940s), gold rush (1970 – 1990s), national agrarian reform (1960s – 1990s), and more recently advancement of extractivist industries, agribusiness and ranching (2000s - present). The opening of major federal highways such as the BR-230 (Transamazon highway) and BR-163 (Santarém-Cuiabá highway) in the 1960/70s impacted much of the Amazon. However, the new infrastructure and the growing connections to the capitalist market-economy did not influence Brasília Legal until the turn of the twenty-first century [24]. The community is located at a distance from these roadways, on the opposite shore of the Tapajós River – a major tributary of the Amazon River (Fig. 1). In the early 2000s, the expansion of small-scale ranching and agriculture and the presence of forestry industries considerably reduced the isolation of the community as well as the control over their natural resources [22, 25].

### Population and study design

In 1999, there were 110 households in Brasília Legal. The total population was 557 people, with 249 identifying as female and 308 as male [26]*.* By 2010, the community’s population had grown by 33%, with a total of 197 households and 742 people, for a total of 338 females and 404 males [27]. This increase was primarily due to an influx of workers either seeking employment at the logging company, which opened in 2004, or in the service sector (restaurants, shops, etc).

Longitudinal data was collected with 22 families in Brasília Legal in 1999 and again with the same families in 2010. Four of the families who participated in the 1999 study were unavailable for follow-up due to illness, death or work. The 1999 study was carried out for a year [20], while in 2010 the study was conducted for 2 weeks, from September 20th to October 4th, because of field-related logistical constraints in remote areas of the Amazonian rainforest and limited financial resources. We matched this 2010 period with the corresponding dates in the 1999 study, to have comparable data, notably to account for seasonal variations in locally produced and harvested foods.

For both years, we conducted dietary surveys to document daily food consumption (including solids and/or liquids) using the “Food Diary” method [20, 23]. Families filled out a tally sheet that was divided into different food categories: cereals, animal protein (fish, game, beef, pork, poultry), dairy products, vegetables/legumes, tubers/roots, spices, fruits and beverages. Categories were chosen to track the consumption of traditional Amazonian foods as well as industrially farmed meat products and other processed foods. Every day, families indicated which food items they had consumed in the household. In general, women were responsible for completing the diary. Records were verified and collected by researchers every day, and assistance was provided to participants as needed.

In addition to the food consumption data, socio-demographic characteristics of participating household heads were recorded, including their age, education level, and birthplace (origin). Household living conditions and livelihood strategies were also documented (income sources, primary occupation). To qualitatively understand the observed changes in diet, semi-structured interviews were also carried out with all participants. Open-ended, thematic questions were oriented towards perceptions of food and nutrition, household dietary history, including transitions in the consumption of foods produced by the household and purchased products. Broader changes in local socio-economic conditions, such as employment, access to electricity, transportation, and their effect on diet were also discussed with participants. When necessary, primary interview data about local trajectories of change were corroborated with our knowledge and experience of the region, participant observation, or verified by regional experts.

All participants signed an informed consent form and no compensation was provided. This research is in accordance with the guidelines established by the Ethics Committee on Human Research of the Faculty of Health Sciences at the University of Brasília as well as the National Commission on Ethics in Human Research (authorization number 095/08).

### Statistical analyses

To analyze changes in household food intake and socioeconomic characteristics in Brasília Legal from 1999 and 2010, data were analyzed using the statistical program Statview v.5 (SAS Institute, 1998). We compared changes in sociodemographic data among the 22 families between 1999 and 2010 using McNemar’s test for paired nominal data. Recorded food intake profiles were compared using the Wilcoxon nonparametric test for paired samples, adopting *p* < 0.05 level of significance.

## Results

Table 1 outlines the key sociodemographic characteristics of the 22 families who participated in the food surveys conducted in 1999 and 2010. All participants are from the northern region of Brazil and were born in the state of Pará. Although most informants had at least some level of formal schooling at the time of interview, the level of education was higher among women.

**Table 1:** Socio-demographic characteristics of household heads participating in the 2010 food intake survey

Characteristics	Women* (***n*** = 22)	Proportion of participants (%)	Men (***n*** = 19)	Proportion of participants (%)
**Age**				
31–40 years	2	9.1	0	0
41–50 years	8	36.4	6	31.6
51–60 years	5	22.7	6	31.6
60+ years	7	31.8	5	26.3
No Information	0	0	2	10.5
**Education**				
No formal education	0	0	2	10.5
Basic education (1 to 4 years)	5	22.7	8	42.1
Primary school (5 to 8 years)	11	50	6	31.6
Secondary school (9 to 11 years)	6	27.3	2	10.5
No information	0	0	1	5.3

* In 2010, three of the households were women-led (i.e., there was no male household head)

Table 2 compares the livelihood strategies and living conditions of participating families in 1999 and 2010, demonstrating that there were important changes in household assets and infrastructure over a decade. In 1999, few families had access to electricity through household generators. In September 2010, the federal government installed electrical lines, equalizing the supply of electricity to all families in the community. The primary occupation of household heads has evolved over time. Several household heads transitioned to retirement, highlighting the effect of household aging on livelihood strategies. Similarly, fewer respondents reported fishing and farming activities as their primary occupation in 2010 compared to 1999.

**Table 2:** Economic strategies and living conditions of the 22 households that participated in both the 1999 and 2010 food intake surveys. The bold type/asterisk (*) indicates that there is a significant difference between the 2 years

Characteristics	1999		2010		***P***^**#**^
	Total	(%)	Total	(%)	
**Housing material**					
Brick house	10	45.5	15	68.2	0.065
Wooden house	12	54.5	7	31.8	0.065
**Household assets and infrastructure**					
Television	19	86.4	21	95.5	0.24
parabolic antenna	18	81.8	21	95.5	0.12
Radio	13	59.1	21	95.5	**0.0067**
Well drinking water	22	100.0	22	100.0	NS
Electricity	4	18.2	22	100.0	**< 0.0001**
internal bathroom	12	54.5	15	68.2	0.19
**Income sources**					
Social assistance (Bolsa Familia)	0	0.0	8	36.4	**0.0067**
Fishers assistance (Bolsa Pesca)	0	0.0	6	27.3	**0.021**
Pension	1	4.5	10	45.5	**0.0079**
Public servant	9	40.9	9	40.9	0.342
Formal employment	1	4.5	4	18.2	0.186
Small business	5	22.7	8	36.4	0.225

* In 1999, two of the households were women-led (i.e., there was no male household head) and in 2010 this increased to three households, which is associated to the death or emigration of men

# one-tailed McNemar’s test p-value to test whether socio-economic conditions of the 22 households have improved between 1999 and 2010

Overall, household income sources changed over time, with significant increases in the number of families benefiting from public assistance. In the 2000s, poverty alleviation and rural development programs were introduced in Brazil, offering new income sources to households. At the time of the follow-up survey in 2010, about half of the participating households were receiving social benefits from the *Bolsa Pesca* (assistance for rural fishers to sustain their practices) or from *Bolsa Familia* (a conditional cash transfer program that provides a monthly salary to families with school-aged to subsidize household income). Likewise, more than a third of the participating households began receiving benefits from the public pension plan, which provides a monthly salary to qualifying rural workers over a certain age.

A comparison of food intake in 1999 and 2010 shows a significant increase in the consumption of cereal items, such as rice, pasta, cake and corn (*p* < 0.05 for each, Table 3). While no difference in fish consumption was documented, there was a considerable increase in the consumption of beef and frozen (commercially farmed) chicken. When all purchased meats were grouped together, the increase in consumption of this protein source is significant (*p* < 0.0001). On the other hand, intake of local free-range chicken and game meat significantly decreased (*p* = 0.0159, 0.0066 respectively).

**Table 3:** Daily food consumption in 1999 and 2010 of participating families, expressed as the total number of days a food item was consumed out of the 15 days that were surveyed

Food category	1999 (mean ± SD)	2010 (mean ± SD)	Trend^**a**^	Wilcoxon Test (***p***-value)
**Cereals**				
Rice	12.4 ± 3.6	14.7 ± 0.7	**↑**	**0.0019**
Bread	12.7 ± 0.6	12.0 ± 2.5	**-**	0.4441
Pasta	1.8 ± 2.5	4.3 ± 4.0	**↑↑**	**0.0277**
Cookies/crackers	1.9 ± 2.9	2.0 ± 1.8	**-**	0.6791
Cake	0.4 ± 0.7	1.7 ± 2.2	**↑↑↑↑**	**0.0356**
Corn	0.5 ± 1.3	1.7 ± 2.0	**↑↑↑**	**0.0061**
**Animal protein**				
Carnivorous fish	4.6 ± 3.1	3.5 ± 3.6	**-**	0.1730
Omnivorous fish	4.4 ± 2.8	6.1 ± 2.9	**-**	0.0853
Herbivorous fish	4.5 ± 3.2	4.4 ± 2.2	**-**	0.8789
Beef	4.0 ± 2.4	8.7 ± 3.4	**↑↑**	**0.0001**
Local free-range chicken	1.0 ± 1.3	0.2 ± 0.5	**↓↓↓↓↓**	**0.0159**
Frozen farmed chicken	0.4 ± 0.8	2.8 ± 2.6	**↑↑↑↑↑**	**0.0006**
Game meat	4.5 ± 2.6	2.6 ± 2.5	**↓**	**0.0066**
Purchased meats^b^	4.4 ± 2.4	9.5 ± 3.1	**↑↑**	**< 0.0001**
Eggs	2.7 ± 2.1	5.5 ± 3.3	**↑↑**	**0.0007**
**Dairy**				
Milk	8.0 ± 5.1	13.4 ± 1.7	**↑↑**	**0.0004**
Butter	12.0 ± 3.9	13.8 ± 2.0	**-**	0.0883
**Vegetables/legumes**				
Total	12.2 ± 2.8	12.7 ± 2.9	**-**	0.8871
Tomato	11.4 ± 3.6	11.0 ± 4.1	**-**	0.7510
Bean	4.2 ± 3.5	8.2 ± 4.2	**↑↑**	**0.0009**
Collard greens	0.6 ± 1.5	2.9 ± 3.9	**↑↑↑↑↑**	**0.0395**
Pepper	1.6 ± 3.0	1.3 ± 2.1	**-**	0.8613
Cabbage	0.04 ± 0.2	2.0 ± 2.8	**↑↑↑↑↑**	**0.0010**
**Roots/tubers**				
Cassava flour	14.7 ± 0.6	14.4 ± 0.7	**-**	0.3139
Cassava	0.0 ± 0.0	1.3 ± 3.8	**↑↑↑↑↑**	**0.0180**
Potato	0.5 ± 1.2	3.0 ± 3.6	**↑↑↑↑↑**	**0.0056**
**Condiments**				
Parsley	7.4 ± 5.6	11.1 ± 3.6	**↑**	**0.0105**
Onion	13.3 ± 2.3	13.3 ± 2.0	**-**	0.8617
Garlic	2.1 ± 4.3	9.4 ± 3.4	**↑↑↑↑**	**0.0002**
Paprika	0.3 ± 1.3	10.2 ± 3.2	**↑↑↑↑↑**	**< 0.0001**
**Fruits**				
Total	8.9 ± 3.8	11.3 ± 3.9	**-**	0.0582
Banana	5.5 ± 4.0	9.2 ± 3.9	**↑**	**0.0075**
*Ingá*	0.04 ± 0.2	0.0 ± 0.0	**-**	0.3287
Orange	0.5 ± 1.0	3.5 ± 3.6	**↑↑↑↑↑**	**0.0018**
Guava	1.2 ± 2.3	0.6 ± 1.8	**-**	0.1834
Mango	1.8 ± 3.3	0.1 ± 0.6	**↓↓↓↓↓**	**0.0323**
*Jambo*	1.4 ± 2.8	0.4 ± 0.9	**-**	0.1330
Watermelon	0.1 ± 0.3	1.8 ± 2.0	**↑↑↑↑↑**	**0.0015**
Avocado	0.5 ± 1.1	0.4 ± 1.0	**-**	0.9492
Pineapple	0.04 ± 0.2	0.5 ± 0.9	**↑↑↑↑↑**	**0.0425**
Apple	0.3 ± 0.8	0.7 ± 1.0	**-**	0.1823
Papaya	0.4 ± 1.3	1.0 ± 1.9	**-**	0.2049
Acerola	0.7 ± 1.2	0.04 ± 0.2	**↓**	**0.0251**
Cashew	2.2 ± 3.2	1.4 ± 2.1	**-**	0.2243
Grape	0.1 ± 0.6	0.3 ± 0.9	**-**	0.4652
Coconut	0.3 ± 0.6	0.1 ± 0.3	**-**	0.1159
**Beverages**				
Coffee	14.3 ± 1.1	13.5 ± 1.9	**-**	0.1261
Natural fruit juice	0.7 ± 2.4	2.1 ± 2.8	**↑↑↑**	**0.0281**
Processed juice	0.04 ± 0.2	2.7 ± 2.7	**↑↑↑↑↑**	**0.0006**
Soft drinks	0.1 ± 0.3	1.6 ± 2.1	**↑↑↑↑↑**	**0.0046**

Bold *p*-values indicate that there is a statistically significant difference in consumption between the 2 years (*p* < 0.05)

^a^Arrows indicate the general trend in daily food consumption over time. The direction of the arrow indicates if the trend is increasing (↑) or decreasing (↓). The number of arrows indicates the amount by which food intake has changed, such that ↑↑ indicates a two-fold increase in consumption, ↑↑↑ indicates a three-fold increase in consumption, etc.

^b^purchased beef, pork, and poultry (frozen chicken) were grouped into a single variable

Overall, families consumed more fruits and vegetables in 2010 than in 1999, especially beans, collard greens and cabbage (*p* = 0.0009, 0.0395, 0.0010 respectively). Among the tubers and roots documented, only intake of cassava and potatoes increased (*p* = 0.0180, 0.0056 respectively). While there is no statistical difference for fruit consumption between survey years when they are grouped together, the analysis of individual fruits does highlight important changes. Specifically, the consumption of bananas, oranges, watermelon and pineapple increased significantly (*p* < 0.05 for each). On the other hand, daily intake of mangos and acerola, two fruits locally harvested, decreased (*p* = 0.0323, 0.0251 respectively). The main streets of the community are lined with large mango trees, however, in 2010 the trees were pruned to their shaft to facilitate the installation of electrical lines. As such, mangos were scarce in the community in the year of the follow-up survey. Coffee was the most consumed beverage in both years and there was no significant change overtime. In 2010, there was an increase in the consumption of natural juice (*p* = 0.0281), processed juices (*p* = 0.0006) as well as soft drinks (*p* = 0.0046).

In addition to the categories in Table 3, the 2010 survey documented consumption of packaged, canned, and cured foods, including bologna, pepperoni, mayonnaise, sour cream, ketchup, canned tuna, sardines and meats, chili sauce, premade seasoning, Sazom® seasoning, condensed milk, tomato paste, instant noodles, vinegar, baking powder, Neston® instant cereal drink, corn starch, corn-based snack products, artificial sweeteners, soybean oil, salt, sugar, tapioca flour, and pizza. These items highlight the diversity of processed foods that were consumed by members of this riverside community in 2010. The daily recorded rate of intake for these foods was, on average, low in surveys and in some cases, there were no records of these foods in 1999. As such, a comparative analysis of these items was not undertaken.

Since the introduction of processed items into diets, participating families reported lower consumption of certain foods, many of local origin. When asked about their historical dietary practices, participants uniformly stated that the following foods were more common when they were children but are now scarce in the region: coconut oil/milk, lard, corn oil, *babaçu* nuts/oil, forest fruits, game meat, local free-range chicken, fresh milk. Participants also indicated that some fish species became less abundant in 2010.

When asked about foods that are being consumed more frequently since childhood, participants reported increases in frozen farmed chicken, purchased meats, beef, non-Amazonian fruits (i.e., apples, grapes and pears), powdered milk, canned meat, premade seasoning, soy oil, pasta, cookies/crackers and soft drinks.

Participants consistently noted that the causes of these dietary changes are rooted in the introduction of new income sources, such as the *Bolsa Familia*, and new employment opportunities on growing, nearby industrial farms or in the lumber industry. They also mentioned improved transportation, greater food access, and the availability of credit plans to purchase foods. Participants also attributed transitions in consumption to land use changes, notably agricultural encroachment on forested areas that decreases the local availability of game meat, as well as overfishing of some species with high commercial values on regional markets.

When informants were asked about the desirability of these dietary changes, 90.1% of families said that the food situation in the community had improved, owing to their increased access to new food items. Only two families felt that the food situation had deteriorated over time, citing the lack of locally produced foods as the cause. In 2010, most participants considered buying food easier than subsistence agriculture and extractivist activities (hunting, fishing and forest gathering), and they valued the higher availability and access to new foods. Nonetheless, many were also aware that environmental degradation has impacted their diets.

## Discussion

Over a ten-year period, food from commercial supply chains became more frequent in household diets in the rural community of Brasilia Legal. Daily consumption of packaged, processed items and foods rich in energy, protein, and sugar significantly increased. At the same time, family meals included fewer traditional, locally harvested foods. These observations parallel trends across Brazil and Latin America of increasing supermarketization and nutrition transitions ongoing since the 1980s [28, 29].

During the study period, the Brazilian Amazon became increasingly integrated into the national and global economies. Publicly-funded development and agrarian programs promoted land-use transitions for small-scale farmers as well as private-sector agribusiness and extractivist industries [30], transforming the social and economic lives of rural riverside communities [18, 21, 31]. There were significant gains in national production and distribution of grains and meats following investments in research, credit and subsidies for the domestic agri-food sector [32]. Regionally, Amazonian cities such as Itaituba and Santarem (Fig. 1) expanded swiftly, with a number of supermarket chains supplying processed and imported foods, enabled by regional and bi-lateral trade agreements that were negotiated the 2000s [32]. At the same time, Brazil’s commodity exports continued to grow, such as GMO soybean and beef exports to the European Union and China [30, 32]. In this context, the rural community of Brasília Legal experienced important shifts in diet.

Between the time of the first survey in 1999 and 2010, improvements in transportation and mobility changed food access in Brasília Legal. The paving of the highway BR-163 (Fig. 1) and more regular river transport options have, on the one hand, allowed urban supermarkets to be supplied with more diverse products, while simultaneously providing rural households with access to cities to purchase food items directly. A similar phenomenon has been observed in the Peruvian Amazon, with the paving the Interoceanic Highway, which was associated with a Westernization of the diet in the rural region of Madre de Dios [33]. More frequently, however, commercial boats took on the role of intermediaries in Brasília Legal, purchasing food in cities and selling them to households or stocking local businesses. While most of these transportation improvements occurred through government-funded programs, in Brasília Legal local road maintenance was being funded by a logging company at the time of the study, demonstrating the underlying influence of private and public sectors on local diets.

New income sources and employment opportunities that increase household purchasing power are likely to have contributed to the observed changes in diet. With higher per-capita revenue, families are able to buy more varieties of food and consume more food overall [34]. Among study participants, national poverty alleviation programs (*Bolsa Familia, Bolsa Pesca*) and rural retirement plans implemented in the early 2000s, became important sources of supplementary income, sometimes doubling revenue of poor households [21]. The logging company also began offering jobs in 2004, not only increasing affluence in the community but also bringing an influx of migrant workers and external capital. In response, the number of small businesses and supply shops grew considerably, increasing the local supply of non-Amazonian fruits, frozen meats, and processed foods. Since the time of the first survey, more families began cattle ranching in Brasília Legal [25], which may also explain the increased beef consumption among participants. Compared to traditional subsistence farming and fishing, ranching is a much more lucrative activity that requires considerably less physical labor, making them attractive options [35].

Household access to electricity in Brasília Legal over the study period could also explain the significant rise in frozen, industrially farmed meat consumption. While families without electricity must consume foods almost immediately, electricity allows items to be stored for later consumption. In 1999, Brasília Legal was not connected to the public power grid, but when the logging company opened in 2004, several families benefited from the generators offered by the enterprise. It wasn’t until September 2010, however, that power lines were installed through the public program *Luz para Todos* (Light for All), supplying electricity to all families in the community. This study was carried out just after installation, so it is expected that equalized access to electricity will further accentuate food shifts in the community.

The decline of traditional Amazonian foods in family meals may be in part attributed to changing livelihoods and lifestyles as well as deforestation, which has impacted the availability of wild game, fruits and nuts. In Brasília Legal, the land area dedicated to cattle ranching doubled between 2001 and 2009, while the forest area dropped from nearly 80% to under 60% coverage [36]. Regional deforestation due to the expansion of agribusiness operations further contributes to declines in local forest biodiversity and species abundance. As one informant put it, “the forest is further away [from the village] now, and it is harder to get fruit, the game is disappearing”. Pasture expansion has also encroached on productive agricultural land in the community and, as a result, locally produced crops have become scarce, which raises important food security concerns.

At the time of the second survey, fewer farmers in the community were producing grain and tuber food staples; however, a significant increase in rice and cassava consumption was documented. Supermarkets sell commercial grains, often produced in southern Brazil, at prices that far outcompete local producers, further promoting the shift away from local production and reinforcing a dependence on purchased items in communities. With the added pressures of climate change on land and water resources, smallholders face additional challenges for producing local crops and harvesting traditional foods.

Overfishing, declining water quality and pollution linked to soil erosion and frequent passage of boats, can also affect the availability of aquatic species and, consequently, local fishing yields. Although another study reported a decrease in fish consumption in the same community between 2000 and 2006 [37], we found no significant difference among participating households between 1999 and 2010. The differences between the two studies suggests that households are not homogeneously affected by declines in availability or access to fish. Participants of this study may have been able to sustain local fish consumption by taking advantage of their ability to explore diverse fish species based on the use of different techniques and fishing gear types [22], or they may have continued to engage in fishing activities for leisure despite working new jobs. In any case, links between resource degradation and abandonment of traditional livelihood activities, such as slash-and-burn farming, fishing and forest gathering, have been reported in the region [21], and related impacts on diet and nutrition are expected.

The increasing consumption of processed foods, often with low nutritional values, suggests that Brasília Legal is experiencing a nutrition transition [4, 18], which has implications for the health and nutritional status of riverside communities. With new varieties of foods and loss of traditional diets, it is expected that the intake of macro and micronutrients is changing. For example, game meat in the Amazon is a key source of micronutrients and associated to higher nutritional status [38], while fish provides essential omega-3 fatty acids that support cardiovascular and visual health [39, 40]. However, consuming fish also exposes people in Brasilia Legal to mercury, with progressive long-term health effects documented in this community since the 1990s due to high exposure levels [37]. Selenium, which is acquired from Brazil nuts collected in forests, is an important antioxidant that also decreases the toxicity of mercury [41]. A recent study in the Peruvian Amazon showed that the adoption of a Western diet in the city, but not in rural communities, was associated with a decrease in selenium intake, which could compound the chronic health effects associated with the nutrition transition [33]. In the current context of decreased quality of diet in the region, it cannot be ruled out that the health effects of previous exposure to mercury and those related to the nutrition transition could be exacerbated by current nutritional deficiencies.

An additional health concern is the increase in dietary caloric intake, as more food is being consumed overall and more processed sugars were introduced into diets [42]. Compounded by new modes of employment and abandonment of traditional livelihood practices, lifestyles in the community became more sedentary, which often leads to reduced expenditure of calories consumed [42, 43]. In a similar case study in the Amazon, Piperata and colleagues [18] found that economic development corresponded to a modest weight gain among adults, especially women. Given that obesity is associated with a variety of chronic illnesses [44], a food transition could have negative impacts on the population, particularly with the limited access to health care services and nutritional information that rural Amazonian communities face.

This study has a number of strengths which are worth underlining. The study reports the data collected in the same families surveyed in 1999 and 2010, providing an opportunity for paired-comparisons to document the changes which occurred within household diets over this period. The team has decades of experience working with this community, which made it possible to create a food diary adapted to the local diet. The mixed-methods approach, by which participant interviews are combined with quantitative data from food diaries, provides an opportunity to develop an in-depth understanding of changes and their underlying drivers. This study also has certain limitations, the main one being that the food diary methodology we used did not collect information about the origin of the specific food items reported being consumed (i.e., produced/harvested by the family or the community, bought at a local business, or bought at an urban supermarket). Another limitation is related to the fact that data was collected at the household level and not the individual level. Food consumption patterns are known to differ according to age and gender [34], so we cannot assume that the changes observed in this study apply to all household members. For example, previous studies in communities experiencing a nutrition transition showed that younger people tended to consume more processed foods and less traditional foods compared to older people [28].

## Conclusion

Over a period of 10 years, there were significant shifts in household diet in the riverside community of Brasília Legal in the Brazilian Amazon. In addition to higher overall consumption of meat, dairy, and cereals, intake of novel food items increased such as sugar-sweetened processed beverages, non-Amazonian fruit varieties, and frozen, farmed meats that are purchased in supermarkets and often imported from elsewhere in Brazil and South America. At the same time, traditional Amazonian foods became less present in diets, including forest fruits and game meats that are associated with local harvesting practices.

Regional development processes driven by planned government programs and private sector involvement appear to be underlying these dietary shifts. Infrastructural improvements saw the construction of roads, enhanced river transportation, and installation of electrical lines, allowing households to gain access to and store supermarket-bought foods. New employment opportunities and subsidized income programs boosted household wealth and power to purchase such products. At the same time, deforestation and expansion of pasturelands have impacted the availability of forest-derived foods around the community, probably contributing to decreased intake of traditional Amazonian dietary components.

While these dietary changes suggest a nutritional transition, given the lack of biometric data, we cannot conclusively diagnose nutritional status. Furthermore, in the Amazon, there is little data with which to compare our results, raising the need for continuous monitoring of community population health by means of longitudinal studies to complement clinical and epidemiological studies. Nonetheless, this novel study provides empirical confirmation that there have been significant shifts in diet, which may present risks to human health.

Our study also highlights potential trade-offs between development programs and population health. Fostering access to affordable energy, alleviating poverty through direct income subsidies, and promoting agrarian development through land-use change seems to be, at the moment, at odds with the maintenance of traditional, healthy dietary habits. Important work has been done to identify food policy areas that need to be addressed to deal with nutrition transition and supermarketization in Latin America, especially in the context of urbanization [28, 43]. Nutrition transitions and the reliance on commercial value chains has been addressed to a much lesser extent in rural areas of Latin America, which are confronted with challenges related to resource extraction, energy production, and other contentious changes in the use and ownership of the local resources. In addition to the need for holistic food policies that address all the components of food systems [45], integrated policies that consider the interactions between different development priorities could greatly contribute to minimizing unintended negative outcomes of economic development in the Amazon. This is of particular importance for sustainable development goals, in which targets and programs interact to create synergies and/or trade-offs at multiple scales [46]. Health and diet education should be a priority for local population to adapt and benefit from the accelerated changes that affect the region [47].

## Data Availability

The datasets analyzed during the current study are available from the corresponding author on reasonable request.
